# Dihydromyricetin Attenuates Myocardial Hypertrophy Induced by Transverse Aortic Constriction via Oxidative Stress Inhibition and SIRT3 Pathway Enhancement

**DOI:** 10.3390/ijms19092592

**Published:** 2018-08-31

**Authors:** Yun Chen, Hui-Qin Luo, Lin-Lin Sun, Meng-Ting Xu, Jin Yu, Lu-Lu Liu, Jing-Yao Zhang, Yu-Qin Wang, Hong-Xia Wang, Xiao-Feng Bao, Guo-Liang Meng

**Affiliations:** 1 Department of Pharmacology, School of Pharmacy and Key Laboratory of Inflammation and Molecular Drug Target of Jiangsu Province, Nantong University, Nantong 226001, China; cytina1012@163.com (Y.C.); luohuiqin23@163.com (H.-Q.L.); linlin_sun1991@sina.com (L.-L.S.); xumengting0222@163.com (M.-T.X.); yujin_yancheng@163.com (J.Y.); guiqulaixi214@163.com (L.-L.L.); zjingyao1994@163.com (J.-Y.Z.); wangyuqin@ntu.edu.cn (Y.-Q.W.); whxyc1711@163.com (H.-X.W.); baoxi@ntu.edu.cn (X.-F.B.); 2 School of Medicine, Nantong University, Nantong 226001, China

**Keywords:** dihydromyricetin, myocardial hypertrophy, oxidative stress, sirtuin 3

## Abstract

Dihydromyricetin (DMY), one of the flavonoids in vine tea, exerts several pharmacological actions. However, it is not clear whether DMY has a protective effect on pressure overload-induced myocardial hypertrophy. In the present study, male C57BL/6 mice aging 8–10 weeks were subjected to transverse aortic constriction (TAC) surgery after 2 weeks of DMY (250 mg/kg/day) intragastric administration. DMY was given for another 2 weeks after surgery. Blood pressure, myocardial structure, cardiomyocyte cross-sectional area, cardiac function, and cardiac index were observed. The level of oxidative stress in the myocardium was assessed with dihydroethidium staining. Our results showed that DMY had no significant effect on the blood pressure. DMY decreased inter ventricular septum and left ventricular posterior wall thickness, relative wall thickness, cardiomyocyte cross-sectional areas, as well as cardiac index after TAC. DMY pretreatment also significantly reduced arterial natriuretic peptide (ANP), brain natriuretic peptide (BNP) mRNA and protein expressions, decreased reactive oxygen species production and malondialdehyde (MDA) level, while increased total antioxidant capacity (T-AOC), activity of superoxide dismutase (SOD), expression of sirtuin 3 (SIRT3), forkhead-box-protein 3a (FOXO3a) and SOD2, and SIRT3 activity in the myocardium of mice after TAC. Taken together, DMY ameliorated TAC induced myocardial hypertrophy in mice related to oxidative stress inhibition and SIRT3 pathway enhancement.

## 1. Introduction

Myocardial hypertrophy includes physiological hypertrophy and pathological hypertrophy [1]. Sustained pathologic myocardial hypertrophy may lead to congestive heart failure, arrhythmia, and sudden death. It is one of the vital causes of many cardiovascular diseases [2]. The exact mechanism of myocardial hypertrophy has not been elucidated, which might be related to oxidative stress, energy metabolism, hemodynamic factors, neurohumoral factors, cardiovascular autocrine/paracrine regulation, insulin secretion, heredity, and so on [3,4,5,6]. Oxidative stress is the state that redox balance of the cell is broken, which means the reactive oxygen species (ROS) levels exceed the scavenging capacity of the antioxidant defense system [7]. ROS directly or indirectly activate hypertrophy-related signal kinases, transcription factors, and extracellular factors to induce and promote cardiac hypertrophy [8]. Paradoxically, clinical studies have found that the use of antioxidants vitamin C and vitamin E is ineffective or even detrimental to cardiovascular diseases [9], while the detailed reason is unknown.

**Figure 1:**
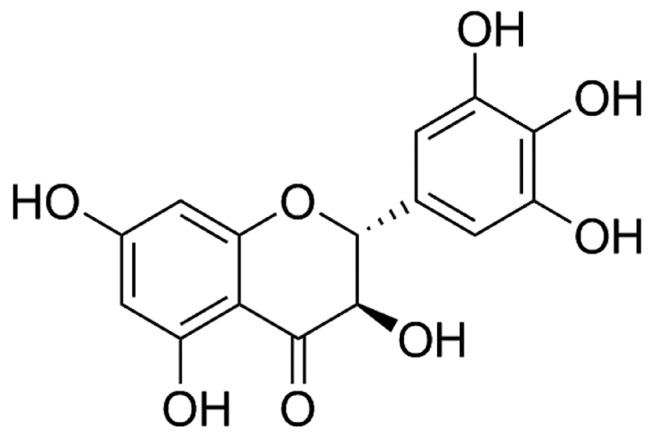
Chemical structure of dihydromyricetin (DMY).

Dihydromyricetin (DMY, the chemical structure of DMY is shown in Figure 1) is a kind of dihydroflavonol flavonoid compound, which is widely found in vine tea [10]. It shows a variety of pharmacological effects including free radical scavenging, anti-oxidation, anti-inflammatory, anti-thrombotic, anti-lipid peroxidation, anti-microbial, liver protection, anti-carcinogenesis, and so on [11,12,13,14,15,16]. Our previous study suggested that DMY pretreatment significantly inhibited angiotensin II (Ang II) induced proliferation of cardiac fibroblasts, decreased collagen I and collagen III levels, suppressed α-smooth muscle actin expression, and attenuated oxidative stress [17]. We also found that DMY inhibited phosphorylation of mitogen-activated protein kinases but increased phosphorylation of endothelial nitric oxide synthase to antagonize Ang II-induced cardiomyocyte hypertrophy [18]. However, whether DMY attenuated myocardial hypertrophy in vivo is unknown.

Sirtuin 3 (SIRT3), a member of the sirtuins’ family, is regarded as the key mediator of mitochondrial biogenesis, and is of great importance on alleviating oxidative stress related diseases [19,20,21]. Previous study suggested that SIRT3 promoted autophagy by forkhead-box-protein 1 (FOXO1) deacetylation, thereby ameliorating Ang II induced myocardial hypertrophy [22]. It is noteworthy that DMY improved hypoxic hypoxia-induced memory loss via a SIRT3 signal pathway [23]. The latest study indicated that DMY ameliorated nonalcoholic fatty liver disease through a SIRT3-depedent mechanism [24]. These studies suggested that DMY was able to regulate a SIRT3 signal pathway to perform potential protective effects.

However, the effects of DMY on myocardial hypertrophy in vivo are not clear. Additionally, whether the SIRT3 signal pathway is involved in the possible protection against myocardial hypertrophy by DMY remains unknown. In the present study, we explored the role of DMY on myocardial hypertrophy induced by transverse aortic constriction (TAC) and investigated the SIRT3-related signal molecules to elucidate possible mechanisms.

## 2. Results

### 2.1. DMY Had No Significant Effects on Blood Pressure in Mice after TAC

**Figure 2:**
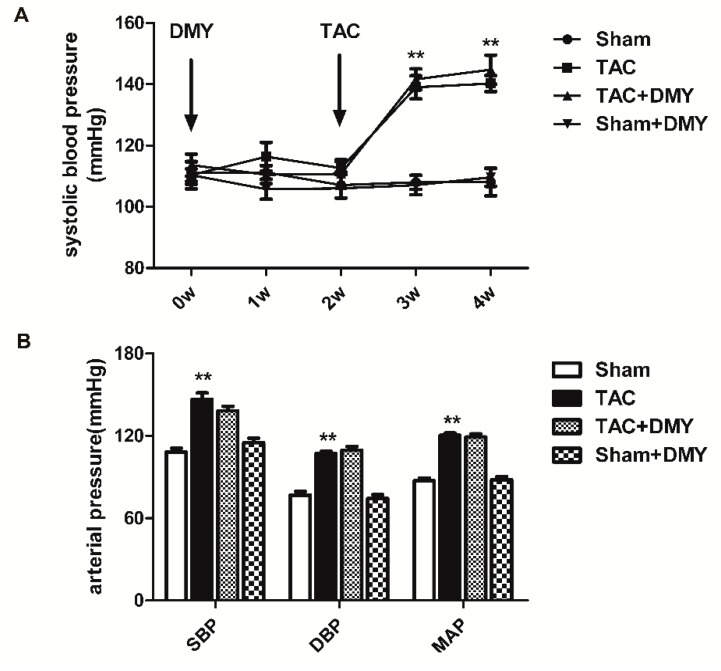
Effect of DMY on blood pressure in mice after transverse aortic constriction (TAC). Male C57BL/6 mice were given DMY (250 mg/kg/day) or carboxymethylcellulose (CMC) (0.5%) by gavage for 2 weeks followed by TAC or sham operation. Then DMY was administered for another 2 weeks. (**A**) Systolic blood pressure (SBP) was measured by tail-cuff method weekly; (**B**) SBP, diastolic blood pressure (DBP) and average mean artery pressure (MAP) were measured via carotid artery cannulation 2 weeks after TAC. ** *p* < 0.01 versus Sham (*n* = 8).

DMY (250 mg/kg/day) was administrated by gavage for 2 weeks followed by TAC. Then DMY was given for another 2 weeks. No mice were dead after TAC operation in our study. For systolic blood pressure (SBP) measured by non-invasive tail-cuff method, the two-way Analysis of Variance (ANOVA) revealed significant effects for TAC, but not for drug treatment or for TAC × drug treatment interaction. For SBP, diastolic blood pressure (DBP) and average mean artery pressure (MAP) measured by invasive artery catheterization, the two-way ANOVA indicated significant effects for TAC, but there were no marked effects for drug treatment or for TAC × drug treatment interaction. Post hoc analysis showed that there was no significant difference in SBP in mice of all the groups at the beginning of experiments. SBP measured by both non-invasive tail-cuff method increased after TAC. SBP, DBP, and MAP measured by invasive artery catheterization also elevated significantly after TAC surgery. However, DMY pre-treatment had no significant effects on both non-invasive and invasive blood pressure (Figure 2).

### 2.2. DMY Improved Myocardial Structure in Mice after TAC

**Figure 3:**
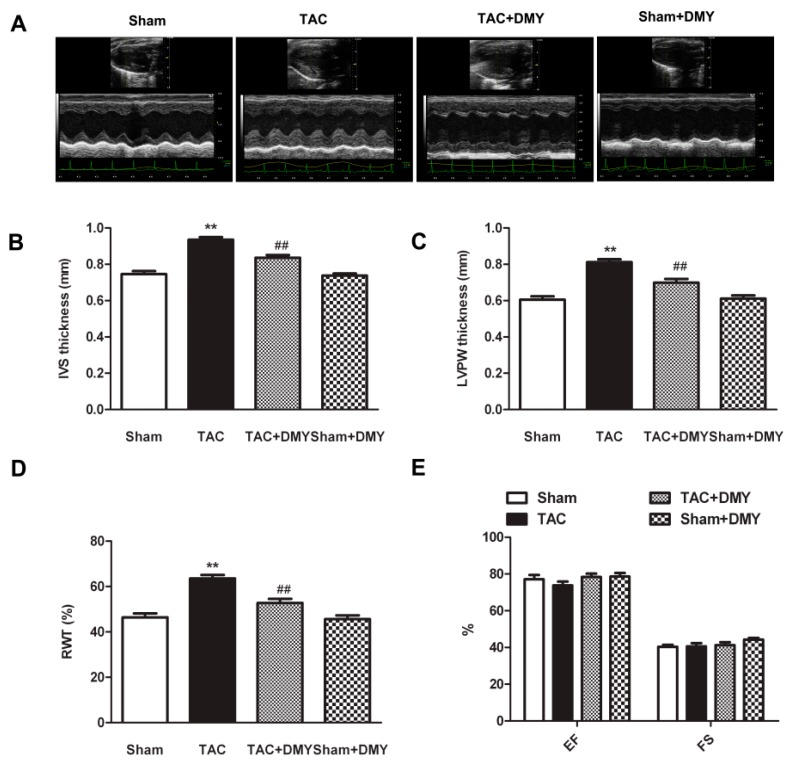
Effect of DMY on myocardial structure in mice after TAC. (**A**) Representative M-mode echocardiograms were shown; (**B**–**E**) Inter ventricular septum (IVS) thickness, left ventricular posterior wall (LVPW) thickness, relative wall thickness (RWT), ejection fraction (EF) and fraction shortening (FS) were quantified by echocardiography. ** p < 0.01 versus Sham, ^##^ *p*< 0.01 versus TAC (*n* = 8).

To determine the effect of DMY on myocardial structure and cardiac function after TAC, we compared the myocardial structure in each group with echocardiography. For the thickness of inter ventricular septum (IVS) and left ventricular posterior wall (LVPW) as well as relative wall thickness (RWT), the two-way ANOVA showed marked effects for TAC, drug treatment and TAC × drug treatment interaction. However, for ejection fraction (EF) and fraction shortening (FS), there were no significant effects for TAC or drug treatment or TAC × drug treatment interaction. Post hoc analysis revealed that mice with DMY pre-treatment exhibited considerable improved myocardial structure, as IVS, LVPW, and RWT increased significantly after TAC (Figure 3A–D). There was no significant difference of ejection fraction (EF) and fraction shortening (FS) in each group (Figure 3E). Additionally, no significant alternation on myocardial structure and cardiac function were found in DMY-treated sham mice (Figure 3).

### 2.3. DMY Reduced Cardiomyocyte Cross-Sectional Area and Cardiac Index in Mice after TAC

**Figure 4:**
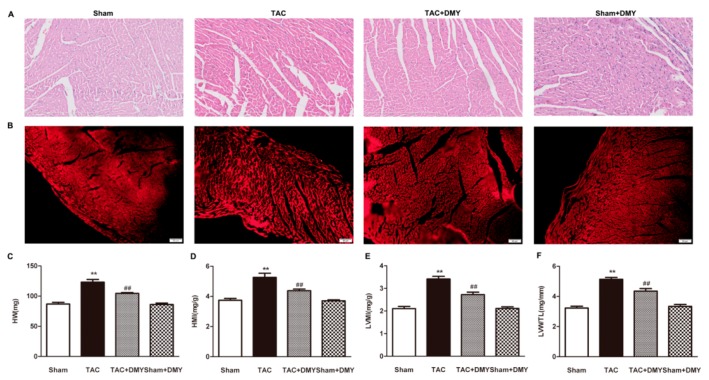
Effect of DMY on cardiomyocyte cross-sectional area and cardiac index in mice after TAC. (**A**) Representative images of hematoxylin and eosin (HE) staining of the myocardium were shown. Bar = 300 μm; (**B**) Representative images of wheat germ agglutinin (WGA) staining of the mice myocardium were shown. Bar = 50 μm. (**C**–**F**) Heart weight (HW), heart mass index (HMI), left ventricular mass index (LVMI) and LVW/TL were calculated. ** *p* < 0.01 versus Sham, ^##^ *p* < 0.01 versus TAC (*n* = 8).

Myocardial tissue sections with hematoxylin and eosin (HE) staining were observed under the microscope (Figure 4A). Wheat germ agglutinin (WGA) staining was also used to measure the cross-sectional areas of cardiomyocytes. Compared with the sham group [(205.4 ± 23.1) μm^2^], the cardiomyocyte cross-sectional area after TAC [(317.9 ± 27.6) μm^2^] increased significantly, which was significantly suppressed by DMY-pretreatment [(254.3 ± 17.23) μm^2^]. There is no significant alternation on cardiomyocyte cross-sectional area in DMY-treated sham mice [(206.6 ± 21.2) μm^2^]. It suggested cell areas were increased after TAC, while DMY reduced the areas markedly (Figure 4B). For cardiac index, including heart weight (HW), heart mass index (HMI) and left ventricular mass index (LVMI), and the ratio of left ventricular weight (LVW) to tibia length (TL), two-way ANOVA showed significant effects of TAC, drug treatment and TAC × drug treatment interaction. Post hoc analysis exhibited a significant increase in HW, HMI, LVMI, and the ratio of LVM to TL in mice after TAC, suggesting that TAC successfully induced myocardial hypertrophy. All these elevated cardiac indexes after TAC were reduced in the DMY group, indicating that DMY attenuated TAC-induced myocardial hypertrophy (Figure 4C–F). No significant alternation on cardiac index was found in DMY-treated sham mice (Figure 4).

### 2.4. DMY Suppressed the Hypertrophic Genes Expression in the Myocardium of Mice after TAC

**Figure 5:**
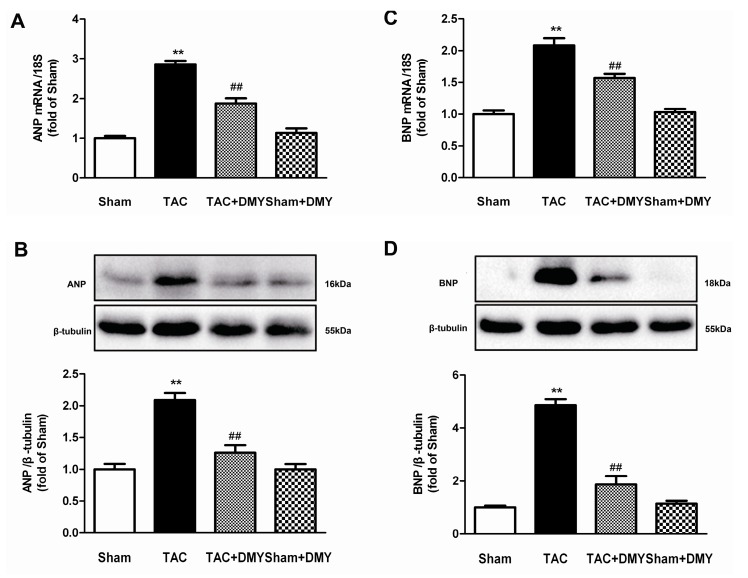
Effect of DMY on hypertrophic gene expression in the myocardium of mice after TAC. (**A**,**B**) Atrial natriuretic peptide (ANP) mRNA and protein expression were respectively quantified by real-time PCR and western blot. (**C**,**D**) Brain natriuretic peptide (BNP) mRNA and protein expression were respectively quantified by real-time PCR and western blot. 18S was used as a housekeeping gene. β-tubulin was used as a loading control. ** *p* < 0.01 versus Sham, ^##^ *p* < 0.01 versus TAC (*n* = 5–8).

In order to further evaluate the effect of DMY on TAC-induced myocardial hypertrophy, atrial natriuretic peptides (ANP) and brain natriuretic peptides (BNP), two hypertrophic genes, expressions were assessed. For ANP and BNP expression at mRNA and protein level, two-way ANOVA revealed significant effects for TAC, drug treatment and TAC treatment interaction. Post hoc analysis showed that TAC markedly increased expression of ANP and BNP at both mRNA and protein level, but this increase could be suppressed by DMY (Figure 5).

### 2.5. DMY Attenuated Oxidative Stress in the Myocardium of Mice after TAC

**Figure 6:**
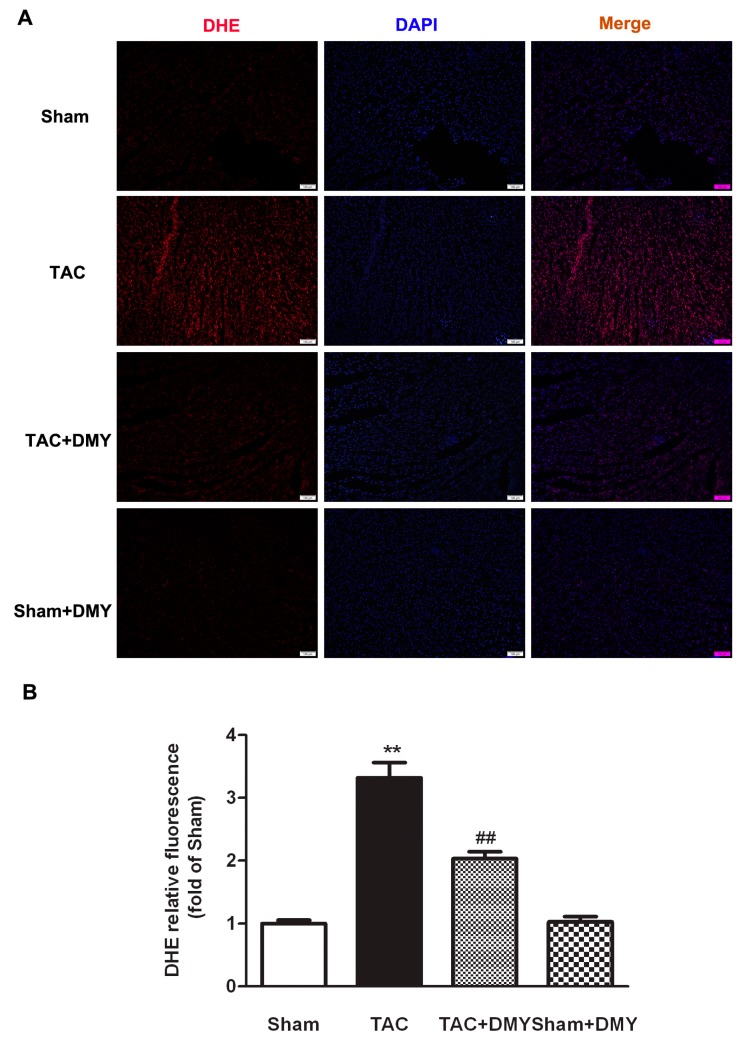
Effect of DMY on oxidative stress in the myocardium of mice after TAC. (**A**) Representative images of dihydroethidium (DHE) staining (Red) of the myocardium were shown. The nuclei were counter-stained with 4’,6-diamidino-2-phenylindole, dihydrochloride (DAPI) (Blue). Bar = 100 μm (Figure 6A); (**B**) Quantification of DHE fluorescence intensity was shown. ** *p* < 0.01 versus Sham, ^##^ *p* < 0.01 versus TAC (*n* = 8).

Oxidative stress plays a vital role in the pathogenesis of myocardial hypertrophy. Dihydroethidium (DHE) staining was performed to verify the effect of DMY on oxidative stress during myocardial hypertrophy. For DHE fluorescence, the ANOVA indicated remarked effects for TAC, drug treatment and TAC × drug treatment interaction. Post hoc analysis indicated that there was stronger DHE fluorescence in the myocardium after TAC, which was attenuated by DMY pre-treatment (Figure 6). These data suggested that DMY inhibited excessive oxidative stress in the myocardium of mice after TAC.

### 2.6. DMY Reduced Myocardial MDA Levels but Enhanced T-AOC and SOD Activity in Mice after TAC

**Figure 7:**
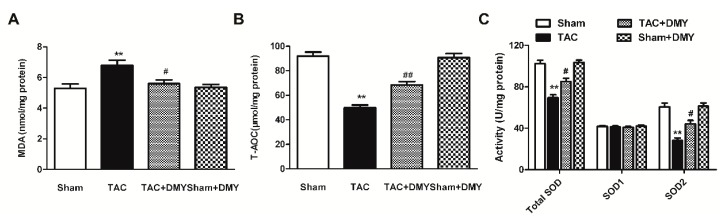
Effect of DMY on myocardial malondialdehyde (MDA) levels, total antioxidant capacity (T-AOC), and superoxide dismutase (SOD) activity in mice after TAC. (**A**) Myocardial MDA levels were detected; (**B**) Total myocardial antioxidant capacity (T-OAC) was measured; (**C**) SOD activity in the myocardium was measured. ** *p* < 0.01 versus Sham; ^#^ *p* < 0.05, ^##^ *p* < 0.01 versus TAC (*n* = 8).

Malondialdehyde (MDA) is one of the most important products of membrane lipid peroxidation, which represents the damage of membrane and the degree of oxidative stress. In the present study, two-way ANOVA revealed significant effect for TAC, drug treatment and TAC × drug treatment on myocardial MDA levels, total antioxidant capacity (T-AOC), activity of superoxide dismutase (SOD) (mainly SOD2 in mitochondria, but not SOD1 in the cytoplasm). Post hoc analysis indicated that TAC increased myocardial MDA levels, which were reduced by DMY pre-treatment (Figure 7A). Our results showed T-AOC and activity of SOD2, but not SOD1, decreased in TAC group, which was restored by DMY pre-treatment (Figure 7B–C). There was no significant change on MDA level, T-AOC, and SOD activity in the myocardium of DMY-treated sham mice (Figure 7).

### 2.7. DMY Increased SIRT3 Expression and Activity in the Myocardium of Mice after TAC

**Figure 8:**
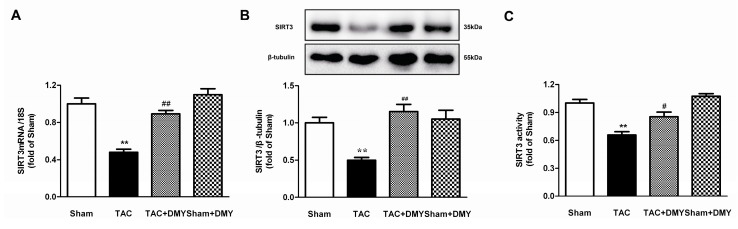
Effect of DMY on sirtuin 3 (SIRT3) expression in the myocardium of mice after TAC. (**A**) SIRT3 mRNA expression was quantified by real-time PCR. 18S was used as a housekeeping gene; (**B**) SIRT3 protein expression was quantified by western blot. β-tubulin was used as a loading control; (**C**) SIRT3 activity was quantified with fluorimetry. ** *p* < 0.01 versus Sham; ^#^ *p* < 0.05, ^##^ *p* < 0.01 versus TAC (*n* = 5–8).

Previous studies have found that SIRT3 has a close relationship with oxidative stress and myocardial hypertrophy [25,26]. To investigate whether SIRT3 is involved in the anti-myocardial hypertrophy of DMY, SIRT3 gene and protein expression in the myocardium were determined. The two-way ANOVA showed significant effects for TAC, drug treatment and TAC × drug treatment on SIRT3 gene and protein expression, as well as SIRT3 activity. Post hoc analysis showed a significant decrease on SIRT3 expression in the myocardium of mice after TAC, which was reversed by DMY pre-treatment (Figure 8A,B). Moreover, SIRT3 activity was reduced after TAC, which was restored by DMY pre-treatment (Figure 8C). No significant change on SIRT3 expression and activity was found in the myocardium of DMY-treated sham mice.

### 2.8. DMY Elevated FOXO3a and SOD2 Protein Expression in the Myocardium of Mice after TAC

**Figure 9:**
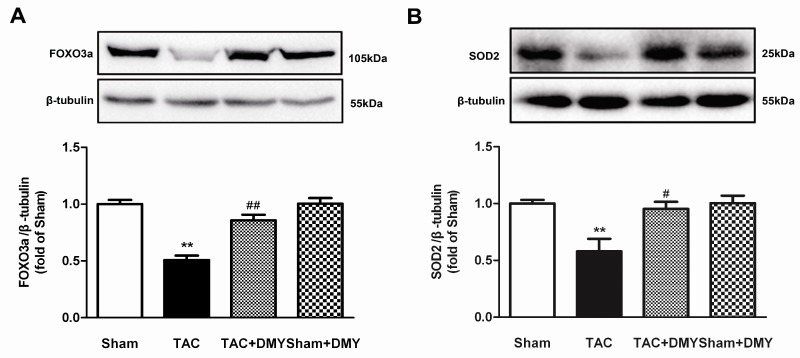
Effect of DMY on forkhead-box-protein 3a (FOXO3a) and SOD2 protein expression in the myocardium of mice after TAC. (**A**,**B**) FOXO3a and SOD2 protein expression were quantified by western blot. β-tubulin was used as a loading control. ** *p* < 0.01 versus Sham; ^#^ *p* < 0.05, ^##^ *p* < 0.01 versus TAC (*n* = 5).

The above results indicated that DMY increased SIRT3 expression during the preventive effects on myocardial hypertrophy. However, the downstream mechanism of SIRT3 involved in this process is not clear. As we know, forkhead-box-protein 3a (FOXO3a) is a transcription factor which suppresses ROS production. SOD2 is also one of the important anti-oxidative stress enzymes to alleviate ROS. More importantly, FOXO3a and SOD2 are important downstream molecules of SIRT3 [19,27]. The two-way ANOVA showed that there were significant effects for TAC, drug treatment and TAC × drug treatment on FOXO3a and SOD2 protein expression. Results of post hoc analysis showed there was a decrease of FOXO3a and SOD2 protein expression in the myocardium of mice after TAC. DMY pre-treatment elevated FOXO3a and SOD2 protein expression (Figure 9). No significant change on FOXO3a and SOD2 expression was found in the myocardium of DMY-treated sham mice.

## 3. Discussion

Pathological hypertrophy is characterized by ventricular wall thickening, myocardial infraction, cardiomyopathy, or structural heart disease caused by long-term hypertension, which is often accompanied by cardiac systolic dysfunction and myocardial interstitial fibrosis, the re-expression of fetal genes such as ANP, BNP, myosin heavy chain β, and so on [28,29,30,31].

DMY, a kind of flavonoid compound, was isolated from stems and leaves of vine tea. Several studies suggested that DMY had multiple cardiovascular protective effects [17,18,32,33,34]. It was found that DMY protected cardiac function, inhibited oxidative stress, reduced inflammatory reaction, alleviated pathological damage, improved mitochondrial function, decreased apoptosis, suppressed autophagy and protected against diabetic cardiomyopathy [32]. DMY reduced serum low density lipoprotein (LDL), interleukin-6 (IL-6), and tumor necrosis factor α (TNF-α) levels in the fat-diet-fed LDLR^−/−^ mice and exhibited anti-atherosclerotic effects [35]. Previous research has demonstrated that DMY alleviated myocardial injury and decreased mortality in doxorubicin-induced myocardial injury in mice [36]. Another study indicated that DMY attenuated atherosclerosis by improving endothelial dysfunction, inhibiting macrophage foam cell formation and ameliorating lipid profiles [37]. Our previous study found that DMY inhibited Ang II-induced cardiomyocyte hypertrophy and myocardial fibroblast proliferation in vitro [17,18]. In this study, we explored the effects of DMY on myocardial hypertrophy in vivo.

It is well documented that blood pressure is one of the most vital factors affecting myocardial hypertrophy [38]. Sustained hypertension is more likely to lead to myocardial hypertrophy. Effective control of blood is an ideal strategy to alleviate myocardial hypertrophy [4]. In order to clarify whether the effect of DMY on myocardial hypertrophy is related to regulation of blood pressure, invasive blood pressure and noninvasive blood pressure were determined. Additionally, our results showed DMY attenuated TAC-induced myocardial hypertrophy without blood pressure lowering effect, which suggested that the protective effect of DMY on myocardial hypertrophy was independent of blood pressure reduction.

Several studies had demonstrated that DMY exerted pharmacological effects via its antioxidant ability. DMY protected neuronal cells against pyruvate-induced oxidative stress in AMP-activated protein kinase/glucose transporter 4 (AMPK/GLUT4)-dependent signal pathway [13]. DMY prevented endothelial cells from hydrogen peroxide-induced oxidative injury by regulating mitochondrial function [39]. DMY also inhibited lipid production and oxidative stress to lessen oleic acid-induced lipid accumulation in L02 cells and HepG2 cells [40]. Previous study proved that DMY suppressed caspase activation but elevated Bcl-2 expression to exhibit a powerful anti-apoptosis effect on osteosarcoma cells [41]. Our previous research demonstrated that DMY suppressed Ang II-induced cardiac fibroblast proliferation via decreasing ROS production [17]. One recent study verified that DMY delayed atherosclerosis process by enhancing the activity of antioxidant enzymes in the liver and aorta [35]. In addition, the improvement on diabetic cardiomyopathy by DMY was also ascribed to oxidative stress inhibition [32]. However, our present study only confirmed that DMY significantly reduced ROS production in global cells but not of the mitochondria of the myocardium, which might be detected in further study. Moreover, we confirmed that DMY significantly decreased MDA levels, suggesting that DMY effectively attenuated oxidative stress in mice after TAC.

The exact mechanisms involved in the antioxidant stress effect of DMY have not been well clarified. We found that decreased SIRT3 expression of the myocardium in mice after TAC was restored by DMY. Sirtuins are highly conserved NAD^+^-dependent deacetylases involved in many cellular processes, including oxidative stress regulation, genomic stability maintaining, and DNA repair [41,42]. Deletion of SIRT3 promoted protein acetylation, cyclophilin D rearrangement, and mitochondrial permeability transition pore opening, thereby resulting in severe oxidative damage [43]. Low expression of SIRT3 resulted in myocardial NAD^+^ depletion, mitochondrial enzyme acetylation, and heart failure, indicating that SIRT3 is pivotal for the maintenance of mitochondrial homeostasis [44,45,46,47,48]. Previous study demonstrated DMY unregulated SIRT3 in HT22 cell in a dose-dependent manner [23]. The latest studies demonstrated that DMY elevated SIRT3 expression to improve hypoxic hypoxia-induced memory and to attenuate the hepatic injury in nonalcoholic fatty liver disease [23,24]. The present results revealed that DMY up-regulated SIRT3 expression, enhanced antioxidant capacity, suppressed oxidative stress, and inhibited myocardial hypertrophy in mice after TAC. These results indicated that elevated SIRT3 expression may be one of the important mechanisms of anti-oxidation during the protective effect of DMY on myocardial hypertrophy. However, the detailed mechanism of how DMY increased SIRT3 expression was unknown. One latest study found that DMY increased SIRT3 expression by activating the adenosine monophosphate-activated protein kinase (AMPK)-peroxisome proliferator-activated receptor-γ coactivator-1 alpha (PGC1α)/estrogen-related receptor-α (ERRα) signaling pathway [24]. Our previous study also found that NaHS increased SIRT3 expression by enhancing PGC-1α expression or increasing activator protein 1 (AP-1) binding activity with SIRT3 promoter [49,50]. It indicated that DMY might also regulate above signaling pathway to increases SIRT3 expression.

SIRT3 is mainly located in mitochondria and deacetylates acetylated mitochondrial proteins, such as acetyl-CoA synthetase, glutamate dehydrogenase, isocitrate dehydrogenase 2 (IDH2), FOXO3a, and SOD2, thereby modulating their activities. SIRT3 increased FOXO3a-dependent gene expression by interacting with daf-16 homolog in mitochondria [51]. Confocal microscopy images clearly showed that there was an interaction between SIRT3 and FOXO3a, indicating that SIRT3 may be a monitoring factor for mitochondrial metabolism. FOXO3a played a vital role in controlling mitochondrial metabolism and redox balance [52]. Environmental stimuli, such as insulin, nutrition, and oxidative stress, regulate longevity genes by altering FOXO activity, protein subcellular localization, DNA-binding properties, and transcriptional activity [53]. FOXO3a and other cellular antioxidant molecules constituted the first line of defense against oxidative stress. FOXO3a was a ROS-sensitive transcription factor that regulated the expression of several important antioxidant genes such as the peroxidase family, glutathione peroxidase, SOD, and so on [54]. FOXO3a regulated the expression of antioxidant enzymes such as SOD2 by deacetylating the acetylation site of the DNA-binding region, regulating its intracellular shift, and binding to DNA. Over-expression of SIRT3 increased the binding force between FOXO3a DNA and SOD2 promoter, thereby increasing the activity of SOD2 promoter [27,55]. The deacetylation effect of SIRT3 on SOD2 increased its enzymatic activity, thereby enhancing mitochondrial ROS scavenging [56]. Our study firstly demonstrated that DMY effectively increased SIRT3 expression and activity during myocardial hypertrophy. Both FOXO3a and SOD2 are important downstream proteins in the SIRT3 signal pathway. Previous study suggested that the HKL-treatment increased SIRT3 levels, which was associated with reduced acetylation of SOD2 [57]. Another study found that overexpression of exogenous SIRT3 protein lowered the acetylation levels of SOD2K68 in diabetic oocytes [58]. FOXO3a was also able to be deacetylated by SIRT3 [59]. Altogether, our present findings suggested that DMY might decrease FOXO3a and SOD2 acetylation to exhibit anti-hypertrophic function, which needs to be elucidated in further study.

In conclusion, DMY attenuates myocardial hypertrophy induced by transverse aortic constriction via oxidative stress inhibition and SIRT3 pathway enhancement in mice. We propose novel evidence that DMY is a potential agent for prevention and treatment of myocardial hypertrophy.

## 4. Materials and Methods

### 4.1. Animals

Male C57BL/6 mice aged 8–10 (8 per group) were provided by Experimental Animal Center of Nantong University (Nantong, China). The experimental procedures were conducted according to NIH Guidelines for Care and Use of Laboratory Animals. The study was approved by the Institutional Animal Ethical Committee of Nantong University (approval no. NTU-20160812, 12 August 2016). The mice were randomized to intragastric administration of DMY (250 mg/kg/day) ((2R,3R)-3,5,7-trihydroxy-2-(3,4,5-trihydroxyphenyl)-2,3-dihydrochromen-4-one, C_15_H_12_O_8_, PubChem identifier: 161557, Standard Center of China, Beijing, China) dissolved in carboxymethylcellulose (CMC, Sinopharm Chemical Reagent Co., Ltd., Shanghai, China) once daily. CMC was used as vehicle for DMY [35]. Two weeks later, anaesthesia was induced with 3% isoflurane in oxygen (3 L/min) and maintained with 1.5% isoflurane. Then mice were subjected to TAC or sham operation.

### 4.2. Transverse Aortic Constriction (TAC)

Mice were anesthetized and artificially ventilated with a respirator and were kept warm on a heating pad. Then left chest of the mouse was opened and the transverse aortic arch was ligated between the innominate artery and the left carotid artery with a 6-0 silk suture ligature tied firmly against a 26-gauge needle, followed by quick withdrawal of the needle to establish a rat model of TAC induced myocardial hypertrophy [60]. Mice in the sham group were underwent the same operation without the constriction.

### 4.3. Blood Pressure Measurement

SBP in mice was monitored by tail-cuff method with a small animal non-invasive blood pressure analysis system once a week (Vistech System, Apex, NC, USA). After echocardiography, a polyethylene catheter filled with heparin saline was inserted into the common carotid artery of anesthetized mice. The pressure transducer was connected with a biological signal acquisition system (MedLab-U/4C501, Nanjing, China) to record carotid SBP, DBP, and MAP.

### 4.4. Echocardiography

Two weeks after surgery, the mice were anaesthetized with isoflurane (1.5%). Myocardial configuration and cardiac function were measured by echocardiography (Visual Sonic Vevo 2100, Toronto, ON, Canada). IVS and LVPW thickness, EF, and FS, left ventricular internal diastolic diameter (LVIDD) were measured. Relative wall thickness was calculated by 2× LVPW/LVIDD.

### 4.5. Cardiac Index Determination

After blood pressure measurement via carotid artery cannulation, the heart was isolated quickly, washed to remove residual blood as much as possible, and dried with a filter paper. Then HW was measured with an electronic balance. LVW including ventricular septum was weighed after atrium and right ventricle had been removed. HW/BW and LVW/BW were calculated, which represent HMI and LVMI respectively. TL from the tibial plateau to the medial malleolus was measured and the ratio of LVW to TL was calculated.

### 4.6. Wheat Germ Agglutinin (WGA) Staining

Heart tissue sections were reconstituted with different concentrations of ethanol (100%, 95%, 85%, 75%, 50% for 1 min respectively), then was washed in distilled water for 1 min. Tissue sections were washed with 0.1M PBS on a shaker 3 times for 5 min. After dry, the sections were put in a dark box, and were incubated with working solution containing WGA-FITC (100 μg/mL; Sigma-Aldrich, St. Louis, MO, USA) and CaCl_2_ (1 mM) for 60 min. After washing carefully for 3 times with PBS, tissue sections were photographed with a fluorescence microscope. Cardiomyocyte area was quantified by morphometric analysis.

### 4.7. Histological Analysis

The myocardium from left ventricular was fixed in 4% paraformaldehyde for 24 h, embedded in paraffin, and cut transversely into 4 μm thickness. Slides were deparaffinized with xylene and rehydrated with graded alcohol and then stained with HE (Beyotime, Shanghai, China). The pathological structure of the myocardium was measured with an inverted phase contrast microscope (Olympus, Tokyo, Japan). Image analysis software was used to calculate the cardiomyocyte cross-sectional area.

### 4.8. Oxidative Stress Evaluation

Production of ROS was evaluated by observing the red fluorescence intensity with DHE (Beyotime, Shanghai, China) staining. In brief, frozen heart tissue was cut into 4 μm sections, followed by DHE (0.2 μM) incubation at 37 °C for 30 min in dark and DAPI incubation at room temperature for 5 min. Intracellular reactive oxygen species, represented as fluorescence, was measured by fluorescence microscopy (Leica, Wetzlar, Germany) at 488 nm excitation and 525 nm emission wavelength. The DHE fluorescence intensity was quantified using Image J software.

As an indicator of lipid peroxidation, levels of MDA in the myocardium were detected using the thiobarbituric acid method (Beyotime, Shanghai, China). T-AOC of the myocardium was measured by the T-AOC Assay Kit with ferric reducing ability of plasma method (Beyotime, Shanghai, China). Concentration of total SOD, SOD1, and SOD2 in the myocardium was evaluated using the WST-1 (2-(4-iodophenyl)-3-(4-nitrophenyl)-5-(2,4-disulfophenyl)-2H-tetrazolium, Beyotime, Shanghai, China) method, in accordance with the manufacturer’s instructions.

### 4.9. SIRT3 Activity

SIRT3 enzymatic activity was assayed using a fluorometric kit (Enzo Life Sciences Inc., New York, NY, USA) according to the manufacturer’s instructions. Protein (40 mg) was incubated at 37 °C or 45 min with specific substrates. Next, 25 mL of developer was added, and samples were incubated for an additional 45 min. SIRT3 activity was measured using a Microplate reader at 350 nm/450 nm.

### 4.10. Quantitative Real-Time PCR

Total RNA from the myocardium was extracted using Trizol reagent (Takara, Kyoto, Japan), and first-stand cDNA was synthesized using PrimeScript™ RT Master Mix Kit (Takara, Kyoto, Japan). Quantitative real-time PCR was performed with SYBR Green (Takara, Kyoto, Japan) Fast qPCR mix (Takara, Kyoto, Japan) with ABI 7500 Real Time PCR System (ABI, Carlsbad, CA, USA). 18S was served as a housekeeping gene. Comparative cycle threshold (CT) (2^−ΔΔ*C*t^) method was used. The primers used are listed as: ANP, sense, 5′-GAGAAGATGCCGGTAGAAGA-3′ and antisense, 5’-AAGCACTGCCGTCTCTCAGA-3’; BNP, sense, 5′-CTGCTGGAGCTGATAAGAGA-3′ and antisense, 5′-TGCCCAAAGCAGCTTGAGAT-3′, SIRT3, sense, 5′-CTGGATGGACAGGACAGATAAG-3′ and antisense, 5′-TCTTGCTGGACATAGGATGATC-3′; 18S, sense, 5′-AGTCCCTGCCCTTTGTACACA-3′ and antisense, 5′-CGATCCGAGGGCCTCACTA-3′.

### 4.11. Western Blot

Proteins were extracted with radioimmunoprecipitation assay (RIPA) buffer (150 mM NaCl, 1% Triton X-100, 1% sodium deoxycholate, 50 mM Tris-HCl, 2 mM ethylenediamine tetraacetic acid, 1 mM phenylmethylsulfonylfluoride, 1 mM dithiothreitol, 10 mM Na_3_VO_4_ and 20 mM NaF, pH 7.5). Homogenates were centrifuged at 4 °C for 15 min, and the supernatant was used for western blot. Proteins of 20–50 μg were separated with sodium dodecyl sulfate polyacrylamide gel electrophoresis (SDS-PAGE, Beyotime, Shanghai, China) and then transferred to a polyvinylidene fluoride (PVDF) membrane (Millipore, Billerica, MA, USA). After blocking with 5% non-fat milk for 2 h at room temperature, the membranes were incubated with anti-SIRT3 (1:1000, Santa Cruz Biotechnology Inc., San Diego, CA, USA), anti-ANP, anti-BNP, anti-forkhead-box-protein 3a (FOXO3a), anti-SOD2 (1:1000, Abcam, Cambridge, UK), or anti-β-tubulin (1:3000, Bioworld Technology, St. Louis, MO, USA) primary antibodies overnight at 4 °C. After washing with TBST, the membranes were incubated with a horseradish peroxidase-conjugated secondary antibody (Santa Cruz Biotechnology Inc., San Diego, CA, USA) for 2 h at room temperature. Finally, the membrane was exposed to enhanced chemiluminescence substrate (ECL, Thermo Fisher Scientific Inc., Rockford, IL, USA) reagent for determination of protein expression.

### 4.12. Statistical Analysis

The data were expressed on mean ± standard error of mean (SEM) and analyzed with two-way ANOVA followed by Bonferroni post-hoc test using Stata 13.0 software (StataCorp LLC, Texas, USA), GraphPad Software (San Giego, CA, USA). A value of *p* less than 0.05 was considered statistically significant.

## Abbreviations

ANP	Atrial natriuretic peptides
BNP	Brain natriuretic peptides
DBP	Diastolic blood pressure
DHE	Dihydroethidium
FOXO3a	Forkhead-box-protein 3a
HMI	Heart mass index
HW	Heart weight
IVS	Inter ventricular septum
LVMI	Left ventricular mass index
LVPW	Left ventricular posterior wall
MAP	Average mean artery pressure
RWT	Relative wall thickness
SBP	Systolic blood pressure
SOD	Superoxide dismutase
T-AOC	Total antioxidant capacity
WGA	Wheat germ agglutinin
